# Geometry-dependent skin effects in reciprocal photonic crystals

**DOI:** 10.1515/nanoph-2022-0211

**Published:** 2022-06-20

**Authors:** Zhening Fang, Mengying Hu, Lei Zhou, Kun Ding

**Affiliations:** Department of Physics, State Key Laboratory of Surface Physics, and Key Laboratory of Micro and Nano Photonic Structures (Ministry of Education), Fudan University, Shanghai 200438, China

**Keywords:** exceptional points, non-Hermitian skin effects, photonic crystals

## Abstract

Skin effect that all eigenmodes within a frequency range become edge states is dictated by the topological properties of *complex* eigenvalues unique in *non-Hermitian* systems. The prevailing attempts to realize such a fascinating effect are confined to either one-dimensional or nonreciprocal systems exhibiting asymmetric couplings. Here, inspired by a recent model Hamiltonian theory, we propose a realistic reciprocal two-dimensional (2D) photonic crystal (PhC) system that shows the desired skin effect. Specifically, we establish a routine for designing such non-Hermitian systems via revealing the inherent connections between the nontrivial eigenvalue topology of order-2 exceptional points (EPs) and the skin effects. Guided by the proposed strategy, we successfully design a 2D PhC that possesses the EPs with nonzero eigenvalue winding numbers. The spectral area along a specific wavevector direction is then formed by leveraging the symmetry of the macroscopic geometry and the unit cell. The projected-band-structure calculations are performed to demonstrate that the desired skin effect exists at the specific crystalline interfaces. We finally employ time-domain simulations to vividly illustrate this phenomenon by exciting a pulse at the center of a finite-sized PhC. Our results form a solid basis for further experimental confirmations and applications of the skin effect.

## 1 Introduction

Band topology has become a key ingredient in physics because it conveys the profound connotation of the eigen-wavefunction of a system exhibiting certain global symmetry [1]. Bulk-edge correspondence endows the robustness of topological edge modes, and a vast myriad of interesting phenomena has then been realized [1–5]. In a Hermitian system, topological invariant roots in a bundle of eigenvectors (wavefunctions) attained by continuously varying the parameters involved in the Hamiltonian [6–10], while the eigenvalues do not carry any topological information but only tell the gap positions [2]. Recent development in *non-Hermitian* theory, however, unveils the eigenvalues can also exhibit topological signatures due that the eigenvalues for a non-Hermitian Hamiltonian naturally live in the complex plane [11]. Such a seemingly straightforward extension makes the eigenvalue winding numbers meaningful, adding another layer to the band topology, which is now called the spectral topology [12–16]. The emerging feature herein is that the band gaps in the complex plane can be classified into two types: the line gap and the point gap [17]. The line gap, literally speaking, is a gap where different energy bands are separated by a line and can be evolved into a traditional band gap through Hermitian flattening. However, the point gap is a singular point surrounded by the energy bands, which is unique in the non-Hermitian system. Since the bands surrounding the singularity always form a closed loop, the area formed by the loop is called a spectral area [17]. Such exciting concepts and the fact that an ideal Hermitian system is usually difficult to realize in real life have made non-Hermitian physics a vibrant field in the past few years [18–25].

Non-Hermitian skin effect (NHSE) is perhaps the most significant phenomenon coming from the nontrivial winding numbers of eigenvalues in the complex frequency (energy) plane [11, 22, 26], [27], [28], [29], [30], [31], [32], [33], [34], [35], [36], [37], [38], [39], [40], [41], [42], [43], [44], [45], [46], [47], [48], [49], [50], [51], [52]. Different from the topological edge modes in a Hermitian system where a few edge states are found within the gap between bulk states, in a non-Hermitian system exhibiting the skin effect, *all* bulk states calculated under the periodic boundary condition (PBC) can collapse to structure edges of the system with the open boundary condition (OBC) imposed [29]. The NHSE was firstly predicted by Yao and Wang in studying a one-dimensional (1D) model system with asymmetric inter-unit couplings [11] and was soon experimentally realized in a 1D photonic system [44–47], inspiring further research in the higher dimensional systems [52–66]. Very recently, Fang et al. predicted that the NHSE can exist in a two-dimensional (2D) non-Hermitian system, as long as the PBC eigenvalues of the system for the wavenumber ***k*** along a particular line in the Brillouin zone (BZ) form a spectral area in the complex plane [52]. An intuitive physics picture is that, as such a condition is satisfied, (complex) eigenfrequencies of a particular ***k*** mode and its specular reflection mode with respect to the crystalline interface must be different, indicating that no standing waves (i.e., bulk modes) can be formed inside the system at these frequencies [17, 52]. In reciprocal systems, such a criterion can only be satisfied on specific paths in the BZ, and thus the desired skin effect can only be found at specific crystalline interfaces of the systems under study, which is now termed the geometry-dependent-skin-effect (GDSE) [52]. From the symmetry point of view, the GDSE obviously requires a mismatch between the geometric symmetry and the lattice symmetry [51, 52].

In this paper, we establish a routine for designing realistic reciprocal photonic systems exhibiting spectral topologies and numerically demonstrate the predicted GDSE in a particular system via full-wave time-domain simulations. Recognizing the intrinsic connections between the nonzero eigenvalue winding numbers of the exceptional points (EPs) and the spectral area, we utilize two pairs of order-2 EPs in a 2D PhC and leverage the symmetry to realize the spectral area and point gaps formed by the complex eigenfrequencies along a non-trivial path in the BZ, which fulfills the criterion of GDSE. We then perform the projected band structure calculations for the corresponding crystalline interfaces with the PBC (OBC) applied along (perpendicular to) the interface and the spatial distributions of all eigenstates within the spectral range of interest clearly show the skin effect. We finally numerically study the time-evolutions of a pulse excited at the center of the photonic structures with trivial and nontrivial interfaces, vividly demonstrating the existence of the predicted GDSE at the nontrivial interfaces of the system.

## 2 Spectral area and point gaps from stable exceptional points

**Figure 1:**
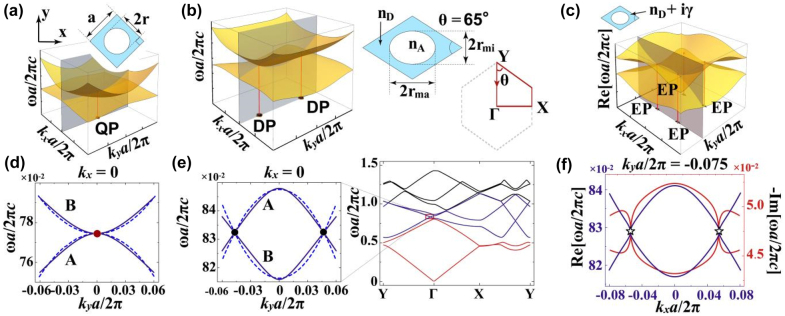
Stable EPs in the 2D reciprocal photonic crystals. (a) Band structure near the Γ point for a square lattice of air holes (*n*_A_ = 1) in dielectric (*n*_D_ = 1.46). Shown in the top is the unit cell with the lattice constant *a* and the hole radius *r* labeled herein. (d) The bands along the *k*_x_ = 0 direction [the gray surface in (a)]. The filled red circles in (a) and (d) highlight the QP at the Γ point. (b) Band structure near the Γ point for an orthorhombic lattice. The rhombic unit cell depicted in the right panel is characterized by the lattice constant *a*, the angle *θ*, and the major (minor) axis of the elliptical hole *r*_ma_ (*r*_mi_). (e) The full band structure along the *Y* − Γ − *X* − *Y* direction is shown in the right-hand side, and the bands forming the DPs are shown in an enlarged view on the left-hand side. The filled black circles in the left panel of (b) and (e) denote the DPs in the Γ − *Y* direction. The A and B in (d) and (e) indicate the band symmetry. (c) The Re(*ω*) surface near the Γ point for the orthorhombic lattice with an additional loss term in the dielectric as *n*_D_ + *i*γ. (f) The values of Re(*ω*) [Im(*ω*)] along the gray surface in (c) are shown by the blue [red] lines. The open black stars in (c) and (f) label the EPs spawned from the DP. The bands in (a), (b), and (c) and the dashed line in (d) and (e) are from the model Hamiltonian, while the solid line in (d), (e), and (f) are calculated by FEM for the Ez polarization. The parameters used are *a* = 1365 nm, *r* = 520 nm, *θ* = 65°, *r*_ma_ = 1.19*r*, and *r*_mi_ = 0.76*r*. The non-Hermitian strength in (c) and (f) is *γ* = 0.1.

We start from designing a 2D reciprocal PhC exhibiting several pairs of stable order-2 EPs. The PhC consists of a square lattice of circular air holes embedded in dielectric, with unit cell shown in the top panel of Figure 1a. Due to the lattice symmetry, there exists a two-fold degeneracy at the Γ point in the band structure. The bottom panel of Figure 1a shows the two bands forming the degeneracy in the *k*_*x*_ − *k*_*y*_ plane calculated by a model Hamiltonian (see Section I in Supplemental Materials for details). A quadratic point (QP) is clearly seen at the Γ point and further confirmed by full wave simulations via the finite element method (FEM), which is shown in Figure 1d. Solid and dashed lines herein, respectively, show the bands along the Γ − *Y* direction calculated by the FEM and the model Hamiltonian. By deforming the square lattice into an orthorhombic lattice depicted in Figure 1b, the QP gives rise to two Dirac points (DPs) in the Γ − *Y* direction which are highlighted by the filled black circles in the left panel of Figure 1b. From the group symmetry point of view, the Γ point belongs the *C*_4v_ (*C*_2v_) point group in the square (orthorhombic) lattice case, and thus the two-fold degeneracy protected by the *C*_4v_ symmetry has been lifted by distorting the unit cell. However, two bands along the Γ − *Y* direction still exhibit orthogonal symmetries, and hence DPs could be formed. The full band structure calculated by FEM is shown in the right panel of Figure 1d and marked by three different colors. The bands in red (black) color are lower (higher) frequency bands, which do not form the DPs and are not focused on in this work. The blue color highlights the bands forming the DPs, and the comparison between the FEM results and the model Hamiltonian is shown in the left panel. Good agreement between two results confirms the existence of Dirac cones in the orthorhombic lattice.

**Figure 2:**
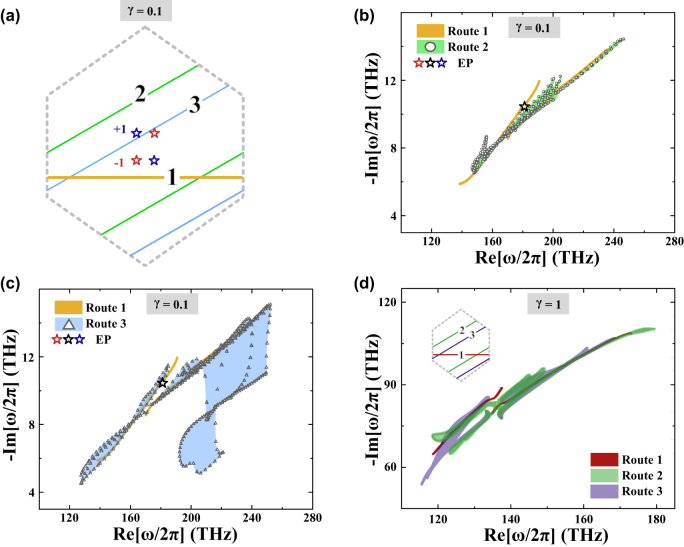
Spectral areas and non-Hermitian band gaps under the PBC. (a) The four order-2 EPs in the first BZ are shown by the open stars with their color displaying the sign of the DN. The solid orange, green, and blue lines show three different routes of interest. (b) The eigenfrequencies along the route-1 and route-2 are shown in the complex frequency plane by the orange lines and open circles, respectively. The profile of the spectral area spanned by the eigenfrequencies along the route-2 is highlighted in green. (c) The eigenfrequencies along the route-3 are shown by the open triangles in the complex frequency plane. The spectral area spanned by the eigenfrequencies along the route-3 is highlighted in blue. The open stars in (b) and (c) denote the EPs, and the non-Hermitian strength in (a–c) is *γ* = 0.1. (d) The spectral areas along the same routes with (a–c) for *γ* = 1. All other parameters are the same with Figure 1.

Once two DPs have been realized, two pairs of EPs are expected to be found in the 2D BZ when non-Hermiticity is present [67–69]. This is because two degrees of freedom (DOFs) are required to stabilize an order-2 EP with nonzero discriminant numbers (DNs) [67]. We assume that the refraction index of the dielectric medium is *n*_D_ + *iγ* with *γ* denoting the dissipative loss. In the case of *γ* = 0.1 which can be realized experimentally, we find that two DPs in Figure 1b give birth to four EPs nearby as shown in Figure 1c. FEM-calculated values of Re(*ω*) [Im(*ω*)] for *k* points on the gray surface (*k*_*y*_ *a*/2*π* = −0.075) are plotted in Figure 1f by the blue [red] lines, from which two EPs marked by open black stars are clearly identified. Note that the non-Hermiticity here does not have any explicit symmetry, and thus each DP only gives rise to a pair of EPs rather than an exceptional ring [68, 70]. To quantify the spectral winding near these EPs, we calculate the DN of each EP *υ*_EP_ by the following equation [51]

(1) $$\begin{array}{ll} \upsilon_{EP} & =\upsilon_{\pm }+\upsilon_{\mp }\\ \upsilon_{\pm \left(\mp \right)} & =\frac{-1}{2\pi }\underset{\Gamma_{EP}}{\oint }dk\cdot \nabla_{k}\arg\left[\omega_{+\left(-\right)}\left(k\right)-\omega_{-\left(+\right)}\left(k\right)\right], \end{array}$$

where Γ_**EP**_ represents the infinitesimal counterclockwise loop in the BZ enclosing an EP with ***k*** being the relevant parameters, and $\omega_{\pm }\left(k\right)$ is the two eigenfrequencies forming the EP. Figure 2a shows the position of four EPs in the first BZ, and the EP in blue (red) indicates its DN is +1 (−1). For a loop encircling the EP, the nonzero DN implies that the eigenfrequencies enclose a spectral area, or equivalently speaking form a point gap, in the complex frequency plane, which is a basic ingredient in the NHSE [51]. As discussed previously, we shall find a set of parallel ***k*** loops along which the corresponding eigenfrequencies form a point gap, and then crystalline interfaces perpendicular to these ***k*** loops must support the GDSE [51]. Therefore, finding such nontrivial ***k*** loops is pivotal in realizing the desired GDSE.

We begin our consideration with the complete *k* loops in the first or extended BZ because otherwise, the eigenfrequency does not come back after travelling one circle. Take the route-1 labelled by the solid orange line in Figure 2a as the first example, which is obviously a complete loop. The FEM-calculated eigenfrequencies along it are plotted in the complex frequency plane by the solid orange lines in Figure 2b. A line gap clearly separates the two bands forming the EP and thus no spectral areas are present, indicating the bands are spectral reciprocal along the horizontal direction. This is because the unit cell possesses the mirror symmetry along both *x* and *y* directions, and thus uncovers the role of symmetry here. The spectral area is expected to be formed in general, but matching the wavevector direction with the unit cell symmetry makes it vanish. Thus, these wavevector directions with zero spectral areas can be treated as trivial ***k*** loops, for instance, the vertical and horizontal directions here. Therefore, the nontrivial ***k*** loops shall be chosen not to possess any symmetry which guarantees spectral reciprocity. With this understanding in mind, we choose two parallel routes which do not exhibit any explicit symmetry and label them as route-2 and route-3 as shown in Figure 2a. The eigenfrequencies calculated by FEM along these two routes are shown by circles in Figure 2b and triangles in Figure 2c, respectively. The spectral area spanned along each route is clearly nonzero, which is highlighted in green and blue. It is then expected that point gaps can be formed when the orientations of ***k*** are parallel to the route-2 and route-3. Comparing with the green region, the spectral regions in blue have been separated into two portions. They are not connected, but the spectral area for each portion is still nonzero (see Section II in Supplemental Materials for details). The same conclusions can be drawn if we increase the non-Hermitian strength to *γ* = 1. As shown in Figure 2d, the spectral area for the route-2 and route-3 is still nonzero but vanishes for the route-1. Hence, whatever the values of *γ*, we expect that GDSE appears at the crystalline interfaces perpendicular to route-2 and route-3 but does not at the interface dictated by route-1. We then consider all the *k* loops other than the vertical and horizontal directions to complete the statement. From the previous argument, these *k* loops shall be nontrivial, but the eigenfrequencies along these loops are not guaranteed to form a point gap solely. However, complementing several *k* segments along the BZ boundary will help a point gap form, and the ensuing GDSE is also expected (see Section II in Supplemental Materials for details).

## 3 Geometry dependent skin effects

**Figure 3:**
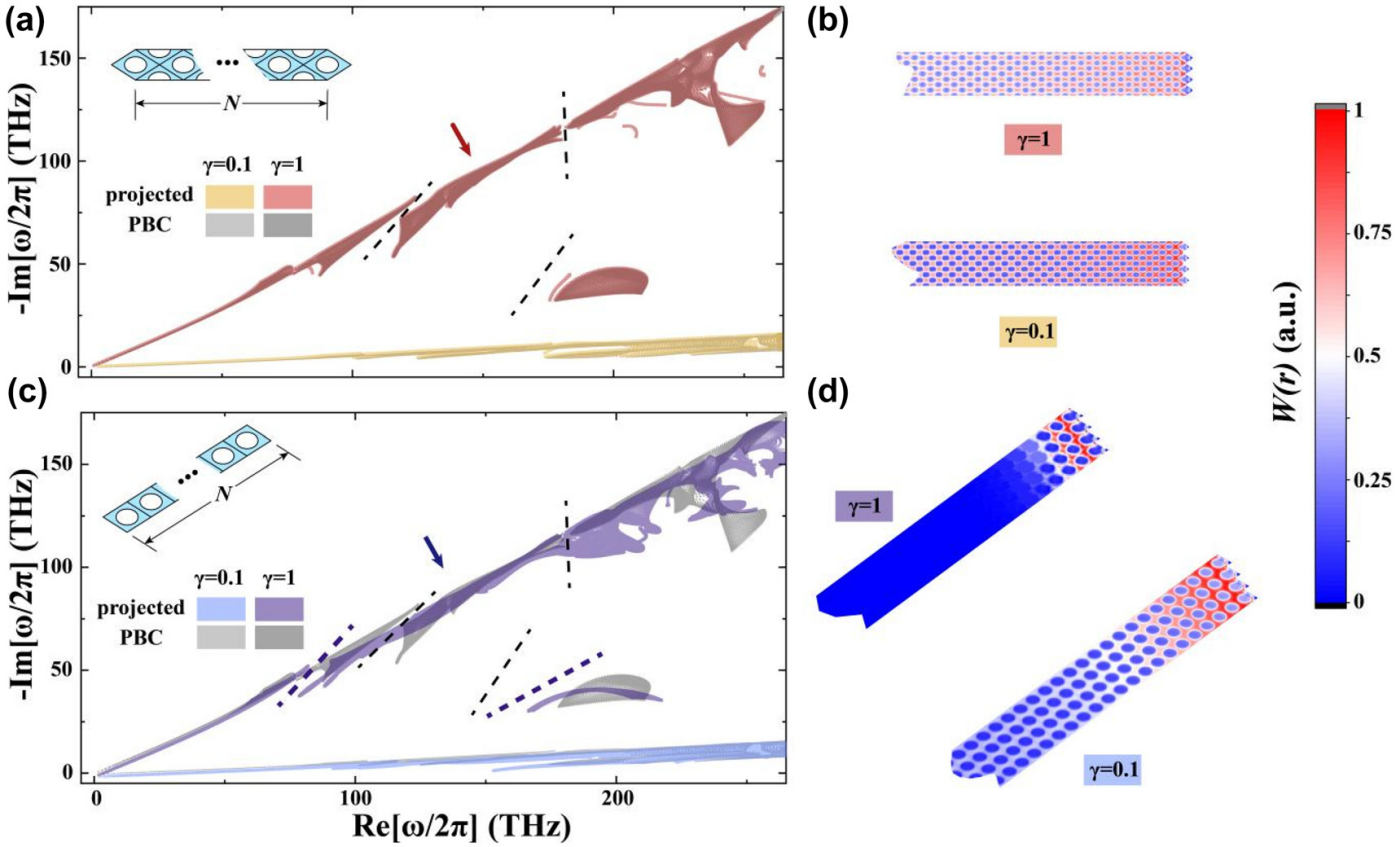
The projected band structure shown in the spectral area view for the crystalline interfaces perpendicular to (a) the route-1 and (c) the route-2/3. The inset in (a) and (c) schematically shows the simulation setup, and the lattice constants are $2a\;\sin\left(\theta /2\right)$ and $a\;\sin\left(\theta \right)$ , respectively. The spectral area with a dark (light) color represents the *γ* = 1 (*γ* = 0.1) case. The gray color denotes the spectral area under PBC, and the black dashed lines denote the line gaps. The spatial distributions of $W\left(r\right)$ corresponding to the configuration (a) and (c) are shown in (b) and (d), respectively. The eigenstates under consideration are indicated by the red and blue arrows. The number of unit cells *N* used in the calculation is 50.

To verify the existence of skin effects for distinct crystalline interfaces, we perform projected band structure calculations to study the structure shown in the inset of Figure 3a and c. The system consists of *N* unit cells in the direction perpendicular to the crystalline interface of interest, and the boundary conditions (BCs) applied to both ends are perfect electric conductors (PECs). The BCs applied along the crystalline interface are periodic boundary conditions (PBCs), which simplify the considered problem into a 1D one and make the analysis traceable [50]. We denote the setup in Figure 3a and c as the horizontal (oblique) configuration which corresponds to the route-1 (route-2 and route-3) in Figure 2a.

Concerning the horizontal configuration (corresponding to a vertical crystalline interface), the calculated eigenfrequencies for all the wavenumbers *k*_∥_ parallel to the interface are highlighted in red (yellow) in Figure 3a for *γ* = 1 (*γ* = 0.1). The (dark) gray region herein shows the eigenfrequencies calculated under PBC. The spectral area calculated within the projected band framework almost coincides with the PBC results, indicating that no significant changes exist. The line gaps denoted by the dashed black lines keep intact, representing that the interface is topologically trivial from the non-Hermitian point of view. However, the spectral area and the related gaps alter dramatically in the oblique configuration, as shown in Figure 3c. We see that the spectral area calculated by the projected setup (OBC) and PBC does not overlap, and the line gaps under PBC (the dashed black lines) disappear in the oblique configuration. Recalling Figures 2d, Figures 3c tells that the point gaps have been formed, and reveals this interface is topologically nontrivial. Such an intrinsic difference between the eigenfrequencies under PBC and the BC-sensitive spectral patterns is a typical feature in the non-Hermitian scenario (see Section II in Supplemental Materials for the comparison between the PBC and OBC results). The consistence of Figures 2 and 3 undoubtedly indicates the two crystalline interfaces are different in nature.

Since the skin effect means that eigenmodes within a frequency range are all localized to the interface, we add the eigenmodes under consideration in an incoherent way as

(2) $$\begin{array}{ll} W\left(r\right) & =\frac{1}{N_{s}}\underset{n}{\sum }w_{n}\left(r\right)\\ & =\frac{1}{N_{s}}\underset{n}{\sum }\left[\varepsilon \left(r\right)|E_{n}\left(r\right){|}^{2}+\mu_{0}|H_{n}\left(r\right){|}^{2}\right], \end{array}$$

where $w_{n}\left(r\right)$ with *n* running from 1 to *N*_s_ denotes the energy density distributions of the *n*th eigenstate [28], $E_{n}\left(r\right)$ and $H_{n}\left(r\right)$ are the electric and magnetic field of the *n*th eigenstate, $\varepsilon \left(r\right)$ is the dielectric constant of the calculated structure, and *μ*_0_ is the permeability of the vacuum. Such a definition smears out the phase information, and thus $W\left(r\right)$ describes the overall distribution characteristic of the considered modes. In the horizontal configuration, Figure 3b shows that $W\left(r\right)$ of the eigenmodes surrounded by the line gaps in Figure 3a distributes uniformly in the bulk region, which illustrates that all eigenmodes here are bulk modes. We then perform the same calculations to study the modes pointed by the blue arrow in Figure 3c, and the $W\left(r\right)$ distributions are shown in Figure 3d. These modes are clearly localized to the interface with strengths exponentially decaying as leaving away from the interface, showing the typical feature of skin modes. Although the decay rate highly depends on the non-Hermitian strength, the fields always decay in an exponential form in Figure 3d (see Section III in Supplemental Materials for details). The comparison between Figure 3b and d nicely demonstrates the GDSE, *i.e.* the crystalline interface corresponding to the route-2 and route-3 supports the skin effects, while the interface corresponding to the route-1 does not. Before proceeding, it is worthy pointing out that our system is Lorentz reciprocal, which already implies the spectral reciprocity. However, the crystalline interface breaks the spectral reciprocity from the macroscopic point of view, generating the desired GDSE (see Section IV in Supplemental Materials for the basic logic flow).

## 4 Time evolution of a pulse

**Figure 4:**
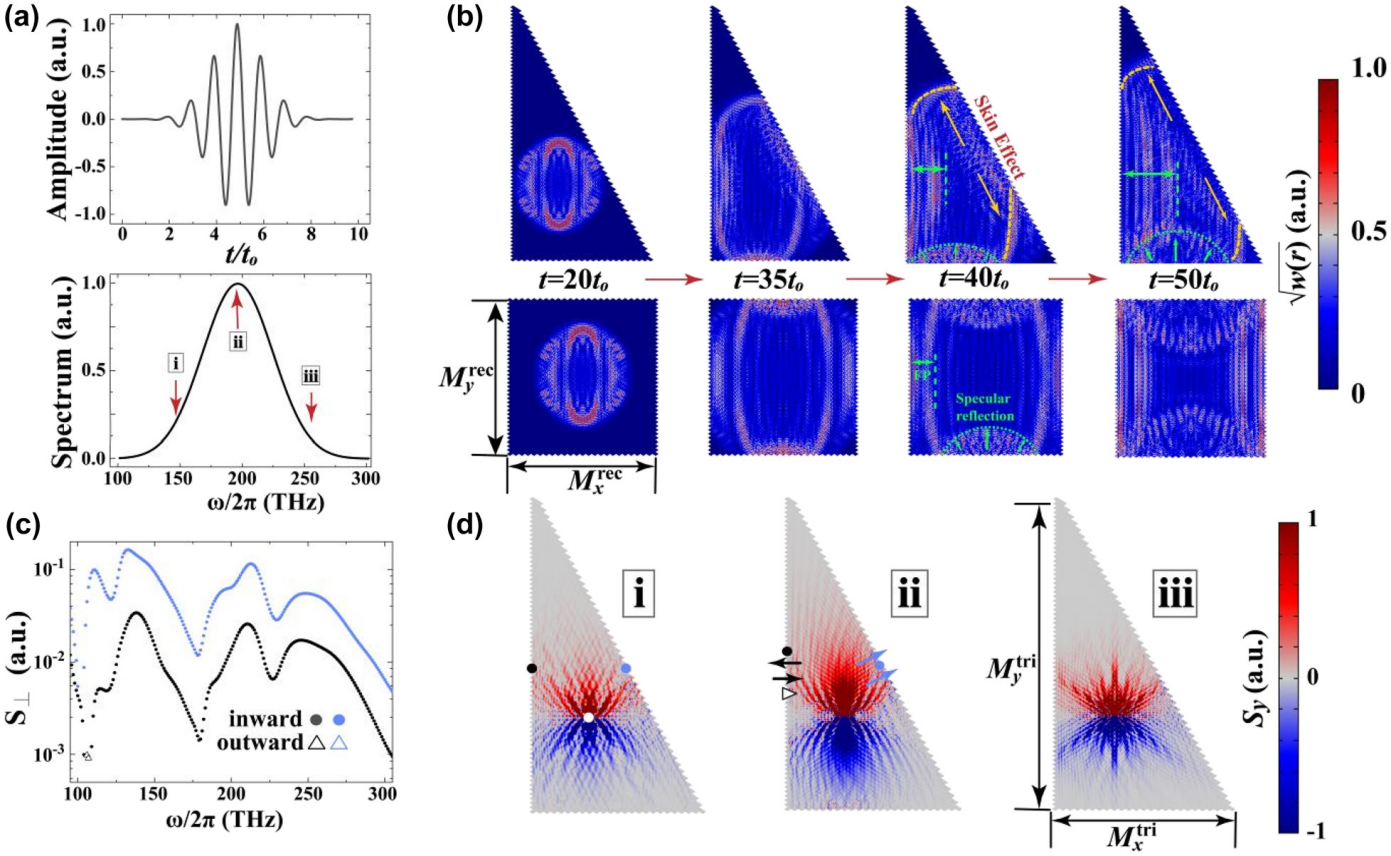
Wave behaviors for the GDSE excited in time and frequency domains. (a) The profile of an input Gaussian pulse in the time domain and its spectrum in the frequency domain. The Gaussian pulse is defined by $J_{\text{z}}=\cos\left(2\pi f_{0}\left(t-1/f_{1}\right)\right)\times \exp\left\{-{\left[\pi f_{0}\left(t-1/f_{1}\right)\right]}^{2}\right\}$ , where *f*_0_ = 195 THz and *f*_1_ = 40 THz are the central frequency and standard deviation, respectively. The time step used in this figure is *t*_0_ = 1/*f*_0_ = 5.1 fs. (b) The calculated time evolution of the Gaussian pulse excited inside a triangle and a rectangle. The color bar is set to square root of the energy density. The dashed green and orange lines, respectively, sketch out the specular reflection and the skin effects. (c) The power flux *S*_⊥_ at the blue and black dots marked in (d) as a function of frequency is highlighted in blue and black. The filled circles (hollow triangles) denote that the averaged power flux is into (away from) the interface. (d) The patterns of *S*_y_ for three frequencies labeled as (i–iii) in the bottom panel of (a). The parameters are *γ* = 0.1, $M_{x}^{\text{tri}}=24$ , $M_{y}^{\text{tri}}=72$ , $M_{x}^{\text{rec}}=25$ , and $M_{y}^{\text{rec}}=40$ .

Considering the excitations of GDSE from the experimental aspect, we construct two finite-sized systems exhibiting triangle and rectangle shapes, respectively, as shown in Figure 4b. While the triangle system has two trivial interfaces and one nontrivial interface which support the GDSE, four interfaces of the rectangle system are all trivial. To probe the GDSE, we excite a Gaussian pulse at the center of two systems and then study its time evolutions. As shown in Figure 4a, the frequency component of the pulse covers the GDSE frequency of interest claimed in Figure 2. Since the pulse is off after 10*t*_0_, we show in Figure 4b the evolution of the pulse beginning from 20*t*_0_. At *t* = 20*t*_0_, the wavefront in both structures does not see the PEC boundaries yet and possesses an elliptical form which is determined by the group velocity of the photonic bands. If nothing nontrivial happens, the waves incident on the edges will experience specular reflections. As time passes by, we see in Figure 4b that the waves touching the vertical edges form the standing wave patterns, showing a strong specular reflection from *t* = 35*t*_0_ to *t* = 50*t*_0_. On the other hand, the waves touching the horizontal edges are also specularly reflected although the wavefront is cylindrical because of the incident pulse. However, when the pulse touches the oblique edge, the waves immediately turn to the upper-left and lower-right direction along the oblique edge, stamped as yellow arrows in Figure 4b (see Section V in Supplemental Materials for the comparison with the Hermitian case). This hints that most of the wave components, no matter directly excited from the source or reflected by the other edges, are deflected along the edge dominantly as long as they are scattered by the interface. This indicates that the pulse is imprinted by the skin effects which are unique to the oblique edge, and hence confirms the GDSE (see movies in Supplemental Materials for details).

To investigate the power flux in the triangular configuration, we perform the continuous wave excitation calculation in the frequency domain. The system is excited by a line source placed at the white point shown in Figure 4d, and we choose two detection points near the edges highlighted by the black and blue circles in Figure 4d. The detection points are far from the line source to ensure that the bulk modes are not recorded. The spectra of the power flux perpendicular to the vertical and oblique edge (*S*_⊥_) are, respectively, shown by the black and blue markers in Figure 4c. We show the norm of *S*_⊥_ in the log scale, while the sign of *S*_⊥_ is dictated by the marker types, since the power flux perpendicular to the interface can be either inward or outward from the edge. Figure 4c tells that the $\left|S_{⊥}\right|$ for the oblique edge is much larger than that for the vertical edge for all the frequencies, and no outward *S*_⊥_ can be found to the oblique edge, hinting negligible reflective waves. In comparison, both inward and outward *S*_⊥_ are clearly seen to the vertical edge, indicating that the waves experience the specular reflection at the vertical interface. The power flux distribution at the frequency (ii) depicted in Figure 4d shows that the power flux bends towards the oblique edge where the GDSE exists. This is consistent with the transport behavior of skin effects in the 1D system [71, 72], further confirming the GDSE (see Section VI in Supplemental Materials for more details of the frequency-domain calculation).

Figure 4 reveals that the skin effects require the propagation of waves to probe, and thus the point gap positions relative to the real frequency axis are crucial in reality. Hence, the averaged magnitudes of Im[*ω*] for a point gap in consideration shall be smaller than its overall real frequency range. Otherwise, the decay process will dominate the wave behavior, which hinders the observation of GDSE. Concerning the calculation in Figure 4, the averaged Im[*ω*] (real frequency range) for the point gap near the EP claimed in Figure 2b and c are about 10 THz (100 THz), making the GDSE observation possible. In other words, the imaginary part of eigenfrequencies cannot be ignored in the transport and excitation scenarios [73].

## 5 Conclusions

To summarize, we propose a strategy for designing a 2D PhC system possessing GDSE. Via the point gaps implied from the order-2 EPs with nonzero eigenvalue winding numbers, the spectral area can be obtained along a particular wavevector direction that does not align with any high symmetry axes of the structure. The crystalline interface corresponding to this wavevector direction supports the skin effect, which has been verified by both the projected band structure calculation and the transport calculation in the time and frequency domain. Our results suggest an alternative way to realize the skin effects in the 2D (3D) system because the order-2 exceptional points (lines) can be found without any prior symmetry requirement in the non-Hermiticity. Thus, the non-Hermiticity can be either from the intrinsic losses or attained by radiative losses of a photonic crystal slab [74]. From the reciprocity theorem point of view, we effectively replace the overall wavenumbers with the wavenumber normal to the interface [66]. Such an approach, together with a myriad of tricks done in the reciprocity of metamaterials, provides a wonderland for manipulating the transport and energy partition of electromagnetic waves [75, 76]. Last but not least, the interplay between the eigenvalue topology of EPs and the spectral topology in the non-Hermitian band structures offers a platform to manipulate the NHSEs in a sophisticated wave system when the multiple order-2 EPs and higher-order EPs are present.

## Supplementary Material

Supplementary Material Details

Supplementary Material Details

Supplementary Material Details

Supplementary Material Details
