# Supplementary material for: Geometry-dependent skin effects in reciprocal photonic crystals
Source: Nanophotonics. 2022 Jun 20;11(15):3447–56. doi: 10.1515/nanoph-2022-0211 (PMC11501693; doi:10.1515/nanoph-2022-0211)
Supplement: Supplementary file 1 — Supplementary Material Details [file j_nanoph-2022-0211_suppl_001.pdf]

Supplemental Materials --- Geometry-dependent skin effects in reciprocal photonic crystals

Zhening Fang, Mengying Hu, Lei Zhou*, and Kun Ding†

Department of Physics, State Key Laboratory of Surface Physics, and Key Laboratory of Micro and Nano Photonic Structures (Ministry of Education), Fudan University, Shanghai 200438, China

*Email: phzhou@fudan.edu.cn

†Email: kunding@fudan.edu.cn

Section I. A two-level Hamiltonian model for the quadratic point and exceptional points

The two-level Hamiltonian which can describe the quadratic point (QP) in the square lattice is read as [S1]

$$H_0 = (\omega_{\mathrm{Q}} + \mu k^2)\boldsymbol{\sigma}_0 + \beta(k_x^2 - k_y^2)\boldsymbol{\sigma}_3 + 2\lambda k_x k_y \boldsymbol{\sigma}_1, \qquad \text{(S1)}$$

where ω_{Q} is the frequency of the QP, $\boldsymbol{\sigma}_i$ $(i = 0, 1, 2, 3)$ are Pauli matrices, and $k^2 = k_x^2 + k_y^2$. The values of β, λ, and μ determine the dispersions of the bands along different directions. Note that the above Hamiltonian works near the Brillouin zone (BZ) center, and we are interested in the bands along the $\Gamma - Y$ direction. Through fitting the finite element method (FEM) results, the parameters used in Figure 1(d) of the main text are $\omega_{\mathrm{Q}} = 2\pi \times 170.2$ THz, $\beta = 1200 \times \left(\frac{a}{2\pi}\right)^2$ THz, $\mu =- 100 \times \left(\frac{a}{2\pi}\right)^2$ THz, and $\lambda = 0$.

Since the Dirac points (DPs) are attained by deforming the square lattice, the Hamiltonian shall have the symmetry breaking terms, which can be written as

$$H_1 = H_0 + H_{\mathrm{b}} = H_0 + \textstyle\sum_{i=1,2,3} \alpha_i \boldsymbol{\sigma}_i, \qquad \text{(S2)}$$

where α_i are the parameters controlling the different ways of breaking the C_{4v} symmetry. α_1 corresponds to changing the relative length of the two lattice vectors, α_2 corresponds to preserving the C_4 rotational symmetry but breaking reflection symmetries, and α_3 corresponds to shearing the lattice which preserves the reflection symmetry with respect to the x and y axes but breaks the C_4 rotational symmetries. For the structure under consideration as shown in Figure S1(a), only α_3 is allowed to be nonzero, and the resulting

eigenvalues are

$$\omega_{\pm} = \omega_{\mathrm{Q}} + \mu k^2 \pm \sqrt{\left(2\lambda k_x k_y\right)^2 + \left[\alpha_3 + \beta\left(k_x^2 - k_y^2\right)\right]^2}. \qquad \text{(S3)}$$

To fit the bands shown in Figure 1(e) of the main text, the parameters are $\omega_{\mathrm{Q}} = 2\pi \times 183.2$ THz, $\beta = 1200 \times \left(\frac{a}{2\pi}\right)^2$ THz, $\mu =- 100 \times \left(\frac{a}{2\pi}\right)^2$ THz, $\lambda = 300 \times \left(\frac{a}{2\pi}\right)^2$ THz, and $\alpha_3 = 6.8 \times \left(\frac{a}{2\pi}\right)^2$ THz.

The Hamiltonian forms for the non-Hermitian system are the same as Eq. (S3), but the parameters herein shall be complex numbers in general. As discussed in the main text, if one parameter or one particular set of parameters become complex and satisfy the pseudo-Hermitian condition, then the DP can give rise to an exceptional ring. However, if all the parameters become complex, then only a pair of EPs can be found near the DP, which is the case shown in Figure 1 of the main text. To describe the behavior near EPs clearly, we set the parameters to $\omega_{\mathrm{Q}} = 2\pi \times (183.2 + 11i)$ THz, $\beta = (1200 + 500i) \times \left(\frac{a}{2\pi}\right)^2$ THz, $\mu =- (100 + 20i) \times \left(\frac{a}{2\pi}\right)^2$ THz, $\lambda = 300 \times \left(\frac{a}{2\pi}\right)^2$ THz, and $\alpha_3 = (6.8 + 10i) \times \left(\frac{a}{2\pi}\right)^2$ THz. The real part of the modeled Hamiltonian is shown in Figure 1(c) of the main text, while the image part is shown in Figure S1(b).

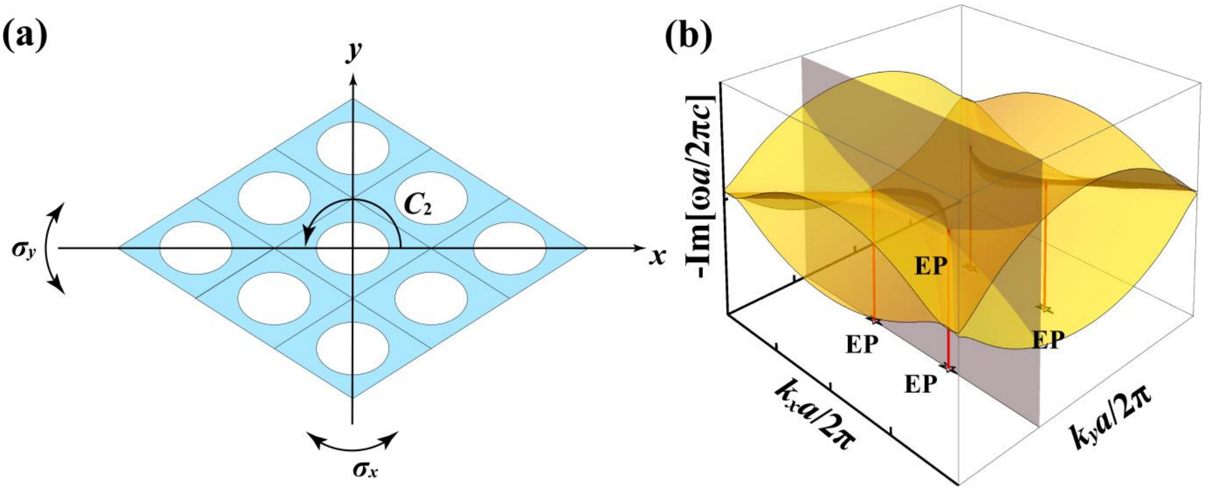

Figure S1. (a) The symmetry operations for the calculated system. (b) The image part of the modeled Hamiltonian. The grey surface is the same with that in Figure 1(c) of the main text located at $k_y a/2\pi =- 0.123$.

Section II. The spectral patterns under periodic and open boundary conditions

As we have shown in the main text, there shall be infinite parallel routes in the reciprocal lattice space perpendicular to the same crystalline interface in the real space. To demonstrate how to choose these routes, we depict in Figure S2 the route-1, route-2, and route-3 under consideration in the extended zone scheme. All routes can form a close loop in the BZ. As shown in Figure S2, the location of the Γ points are: $(0, 0)$ for $\Gamma^{(1)}$, and $\frac{\pi}{\cos(\theta/2)a}\hat{x}+\frac{\pi}{\sin(\theta/2)a}\hat{y}$, $\frac{\pi}{\cos(\theta/2)a}\hat{x}-\frac{\pi}{\sin(\theta/2)a}\hat{y}$, $-\frac{\pi}{\cos(\theta/2)a}\hat{x}+\frac{\pi}{\sin(\theta/2)a}\hat{y}$, $-\frac{\pi}{\cos(\theta/2)a}\hat{x}-\frac{\pi}{\sin(\theta/2)a}\hat{y}$, $\frac{2\pi}{\cos(\theta/2)a}\hat{x}$, $-\frac{2\pi}{\cos(\theta/2)a}\hat{x}$ for $\Gamma^{(2)}$, respectively. The criteria for route-2 and route-3 forming the closed $\boldsymbol{k}$ loops in the BZ are $\overrightarrow{PQ}||\overrightarrow{P'Q'}$, $\overrightarrow{PP'}=\vec{b}_1$, and $\overrightarrow{QQ'}=\vec{b}_1+\vec{b}_2$. By solving the equations, we find that the slope of the route $\overrightarrow{PQ}$ must be fixed while the intercept can be arbitrary.

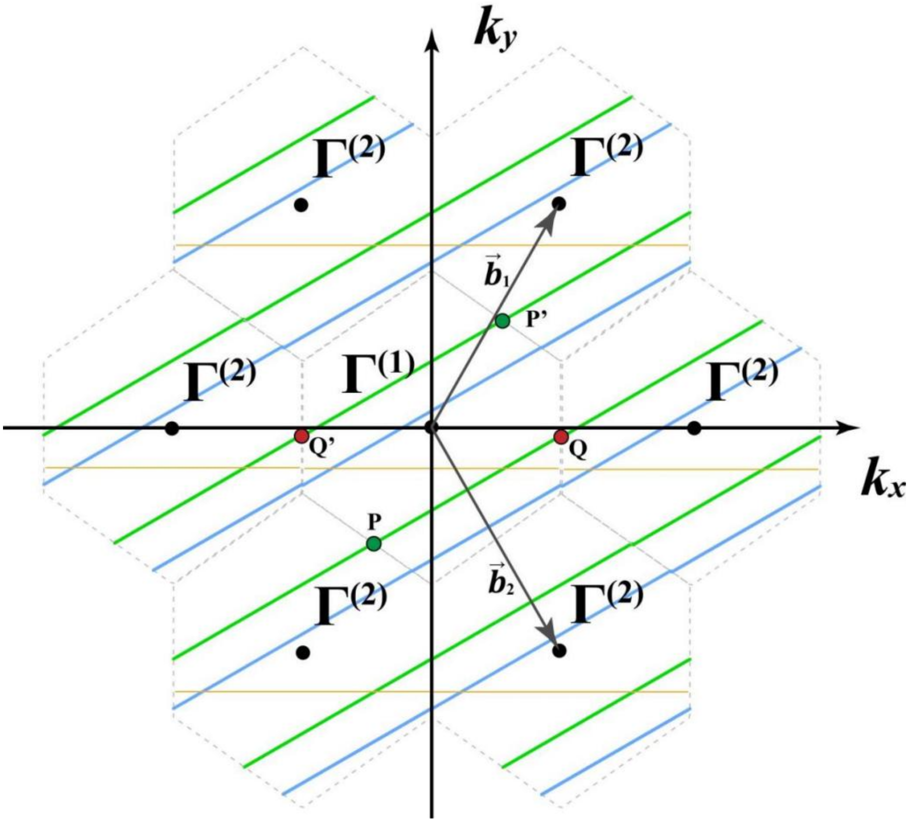

Figure S2. Reciprocal lattice space in the extended zone scheme. Since the point P (Q) and P' (Q') are equivalent, the segment P-Q-Q'-P' (the green route) forms a close loop in the k-space, and the same goes to the blue line. The reciprocal lattice vectors are $\vec{b}_1=\frac{\pi}{\cos(\theta/2)a}\hat{x}+\frac{\pi}{\sin(\theta/2)a}\hat{y}$ and $\vec{b}_2=\frac{\pi}{\cos(\theta/2)a}\hat{x}-\frac{\pi}{\sin(\theta/2)a}\hat{y}$.

Since we only show the spectral winding under the PBC in Figure 2 of the main text, we

provide more evidence in this section. The OBC results for the oblique configuration with $\gamma = 0.1$ are shown with the PBC bands in Figure S3. The PBC bands with a spectral area or a point gap collapse to a line in the OBC calculations, which precisely demonstrates the mechanism of NHSE.

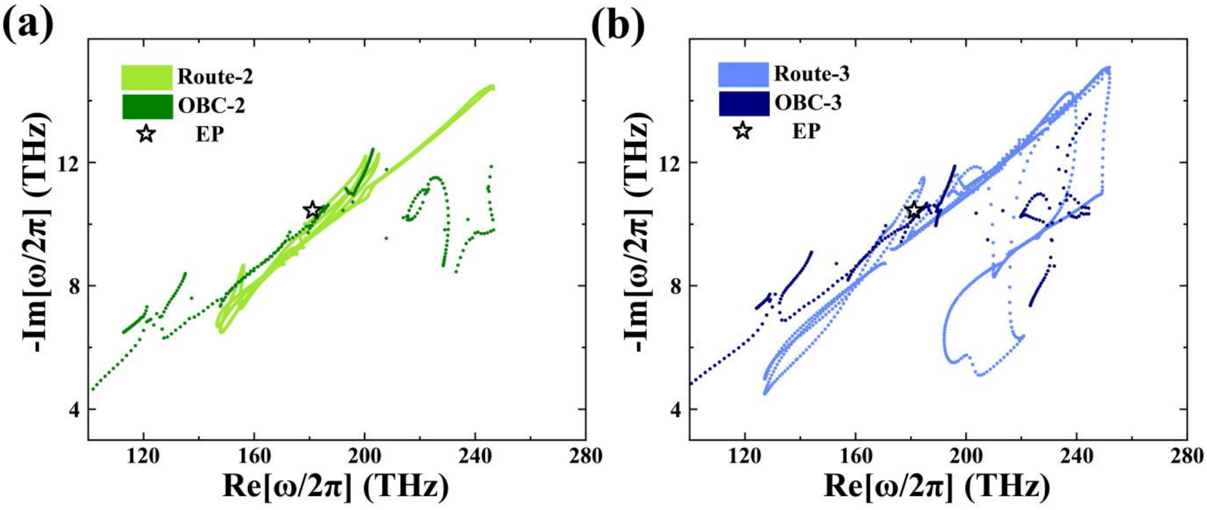

Figure S3. The comparison between spectral patterns calculated under the PBC [same as Figure 2(b-c) in the main text] and the OBC results (darker green and blue dots) for the (a) route-2 and (b) route-3. The black stars denote the EP. The parallel k-vector for OBC-2 (OBC-3) is $k_{||,2} = 0.24 \times \frac{2\pi}{a}$ ($k_{||,3} = 0.06 \times \frac{2\pi}{a}$), which corresponds to route-2 (route-3).

For a holistic view of the bands, the spectral windings for the lowest 8 bands when $\gamma = 1$ are shown in Figure S4. We also calculate all the routes in the reciprocal lattice space with the same slope, finding that once one of the routes can form the spectral area, all the others also can, except the high symmetry axes. Note that only the 1st lowest band does not show a spectral area along the oblique interface, while none of the bands shows the spectral area for the horizontal configuration.

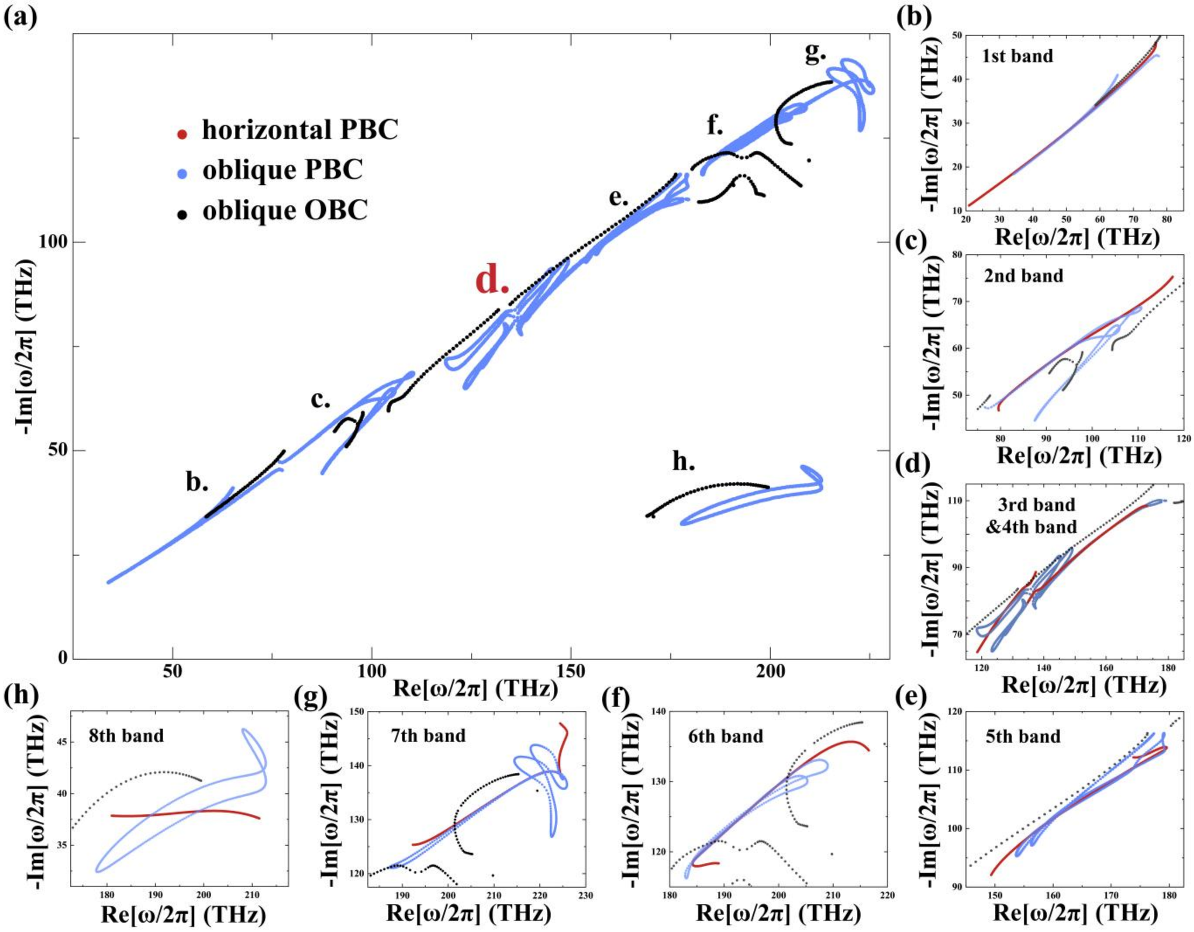

Figure S4. The comparison between spectral patterns calculated under the PBC (blue dots) and OBC (black dots) for the oblique configuration claimed in the main text. The panel (a) shows a full view of the 8 bands, and the enlarged views are shown from panel (b) to (h). The PBC results for the horizontal configuration is also shown by the brown dots in (b-h). Note that (b-c) the lowest 2 bands and (e-h) the 5th to 8th bands are not focused in the work, and (d) the bands containing the order-2 EPs are mostly discussed in the main text. For the OBC results, the PBC is still applied along the crystalline interfaces, and without losses of generality we choose $k_{||} = 0.4 \times \frac{2\pi}{a\sin(\theta)}$. The trajectory of route-1 is $k_y = -0.15G$, and the trajectories of the route-2 and route-3 are $k_y = -0.52k_x + 0.28G$ and $k_y = -0.52k_x - 0.34G$ respectively. All other parameters are the same with Figure 3 in the main text.

As pointed out in the main text, the key to forming non-trivial $\boldsymbol{k}$ loops is the mismatch between symmetries, which means various slopes can be chosen. Figure S5(a) shows another loop in BZ, where the points V and V' are equivalent in the first BZ. The criterion for the specific route is simply derived as: $\overrightarrow{VV'} = \vec{b}_1$. As shown in Figure S5(b), the spectral area in the PBC calculation persists and also collapses to several lines under the OBC. Therefore, the skin effects can still be observed for different non-Hermiticity strengths, as shown in Figure

S5(c). We note here that such a method to obtain the non-trivial closed ***k*** loops is only for the convenience of constructing the crystalline interfaces composed of perfect unit cells. However, the existence of skin effects does not require the chosen ***k*** loops to close solely in the first or extended BZ.

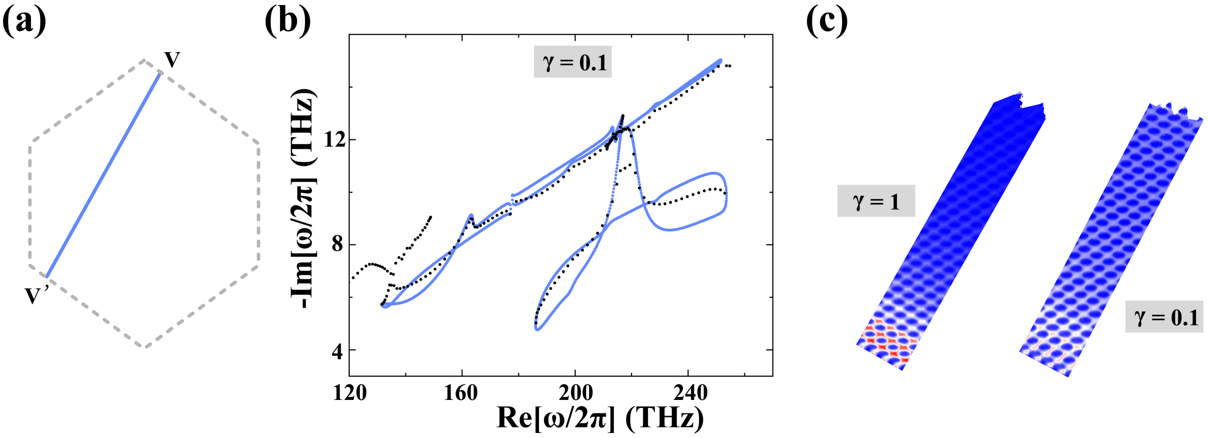

Figure S5. (a) Another chosen route in the BZ where the segment V-V' (the blue route) forms a close loop in the k-space. (b) The spectral areas formed by the chosen route in the PBC and the collapsed bands in the OBC are respectively shown by the blue lines and black dots. (c) The skin effects formed by the bands in (b) with two different non-Hermitian strengths are compared. The parallel k-vector in the OBC is $k_{||} = 0.25 \times \frac{2\pi}{a}$, corresponding to the blue route in (a).

Section III. Numerical verification of geometry dependent skin effects

We have demonstrated in the main text the GDSE based on the one-dimensional setup using in the projected band structure calculations, and the number of unit cells N is fixed to 50. In this section, we increase $N = 100$ to further verify the GDSE, as shown in Figure S6. The distributions of $W(\boldsymbol{x})$ in Figures S6(a) and S6(b) indicate whatever the values of γ, the skin effects always exist in the oblique configuration, but do not in the horizontal configuration. This reveals the geometry-dependent nature, and we shall further verify the asymptotic decay behavior of $W(\boldsymbol{x})$ near the interface to confirm the GDSE. Figures S6(c) and 6(e) show $W(\boldsymbol{x})$ along the black dashed line highlighted in Figures S6(a) and S6(b) for two different values of γ. Besides the geometry-dependent nature, it is also clear that the modes are more likely to localize when γ is larger. To quantify, we use a semi-log plot to $W(\boldsymbol{x})$ near the interface, as shown in Figures 6(d) and 6(f). The linear dependence is clearly seen, indicating an exponential decay of $W(\boldsymbol{x})$. The decay rate is dominated by the values of γ, which reveals the modes are unique to the crystalline interface. This finishes numerical verification of the GDSE.

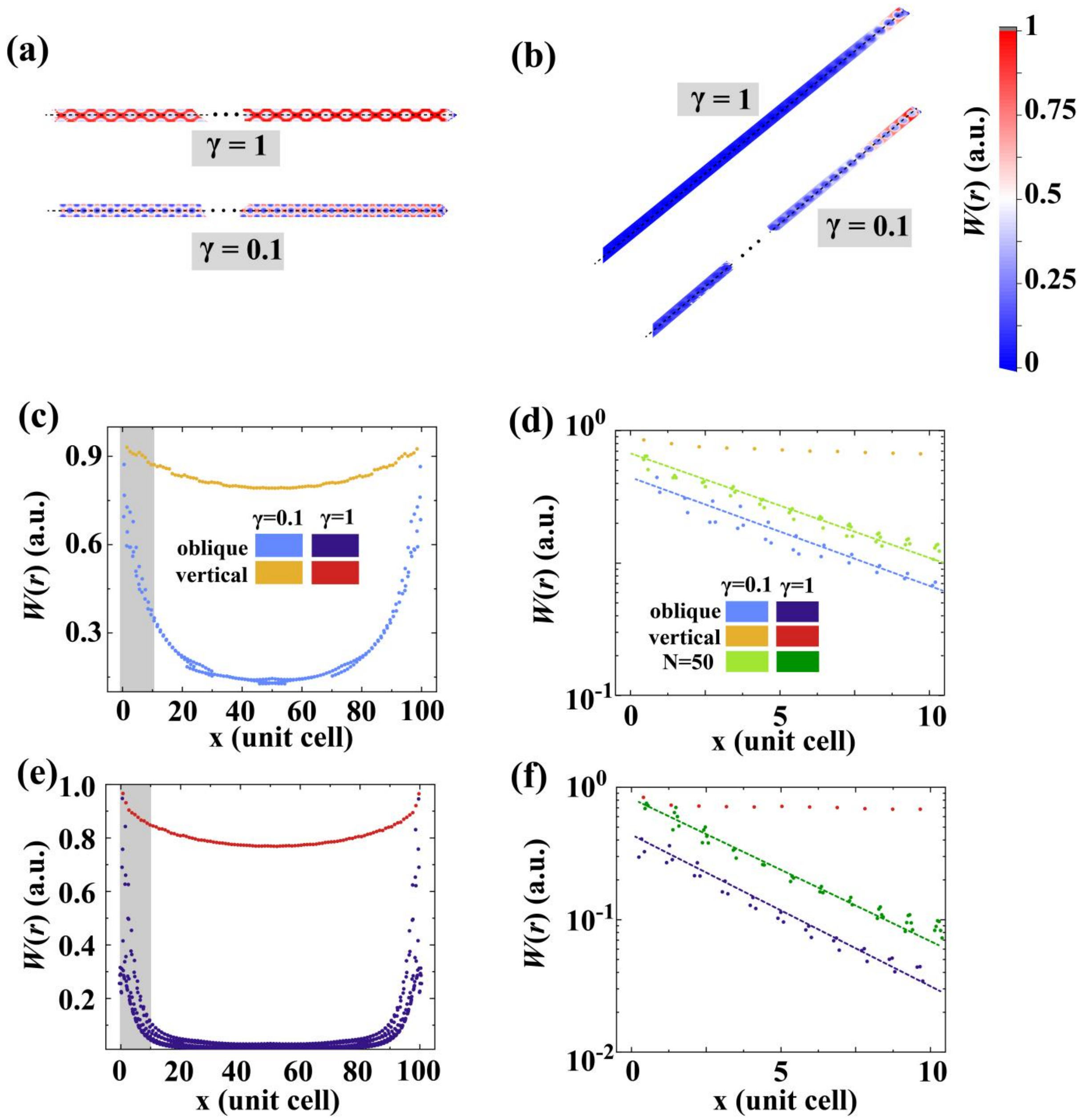

Figure S6. The two-dimensional distribution of $W(\boldsymbol{x})$ for the (a) horizontal and (b) oblique configuration when $N = 100$. The values of $W(\boldsymbol{x})$ as a function of the position away from the interfaces for (c) $\gamma = 0.1$ and (e) $\gamma = 1$ at different interface positions. The semi-log plot of $W(\boldsymbol{x})$ near the interface shown as the grey area for the results in (c) and (e) is shown in (d) and (f), respectively. The blue and purple (orange and red) dots in (c-f) stand for the oblique (horizontal) configuration, while the green and olive dots in (d) and (f) stands for the oblique configuration when $N = 50$, which is the same as the main text. The dashed line shows that the energy density follows the exponential decay from the first unit cell. The fitted curves are $w_{\gamma=0.1}(x) = \exp\{-0.041x\}$ for $\gamma = 0.1$ and $w_{\gamma=1}(x) = \exp\{-0.068x\}$ for $\gamma = 1$.

Section IV. The flowchart of our design strategy

As shown in Figure 2 of the main text, a stable EP in the 2D PhC is guaranteed to have a nonzero DN, while the EP is not a must for the DN. Once the nonzero DN is observed, the symmetry of the unit cell and the overall structure are compared. By mismatching the two symmetries, a closed route in the *k*-space is chosen to ensure the spectral area in the complex frequency plane. With the standing wave argument in the main text, such a spectral area is equivalent to the existence of skin effects upon the corresponding interface.

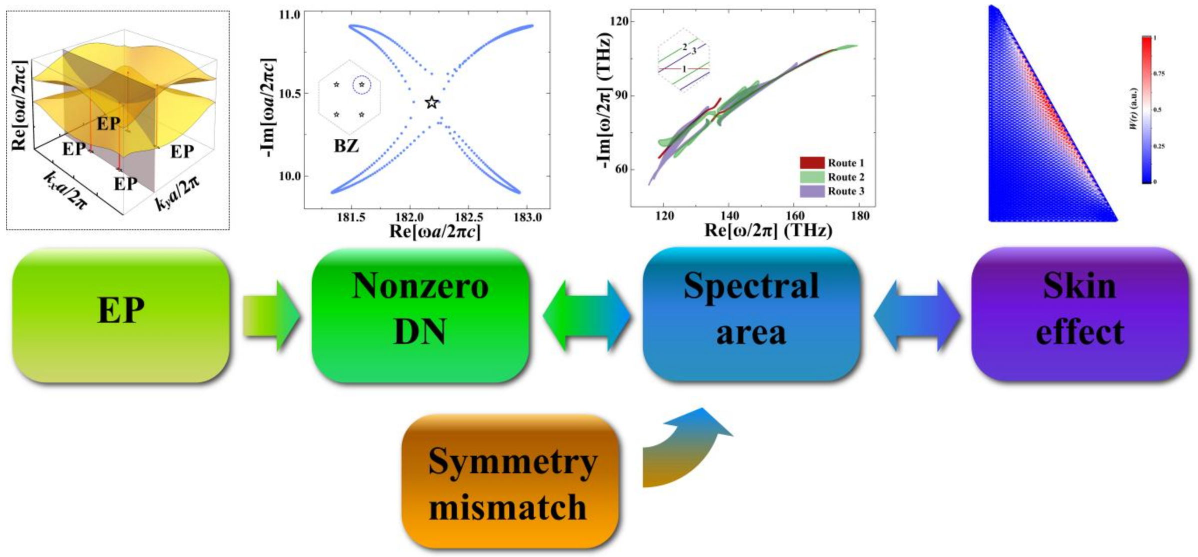

Figure S7. The flowchart of our design. The double arrows denote the equivalency, while the single arrow herein means a sufficient but not necessary condition.

Section V. Comparison with the Hermitian scenario

We have shown the evolution of a pulse inside a triangle and a square for the same value of γ in Figure 4 of the main text to demonstrate the non-trivial crystalline interface. Here, we compare the triangular configuration possessing non-trivial skin effects ($\gamma = 0.1$) with the same configuration without the skin effects by setting $\gamma = 0$. Figure 4(c) of the main text has been duplicated as Figure S8(a), and Figure S8(b) shows the results when $\gamma = 0$. As shown in Figure S8(b), the waves touching the oblique edges show the spectral reflections, while the spectral reflections are not seen in Figure S8(a). This also illustrates that skin effects are found in our non-Hermitian system.

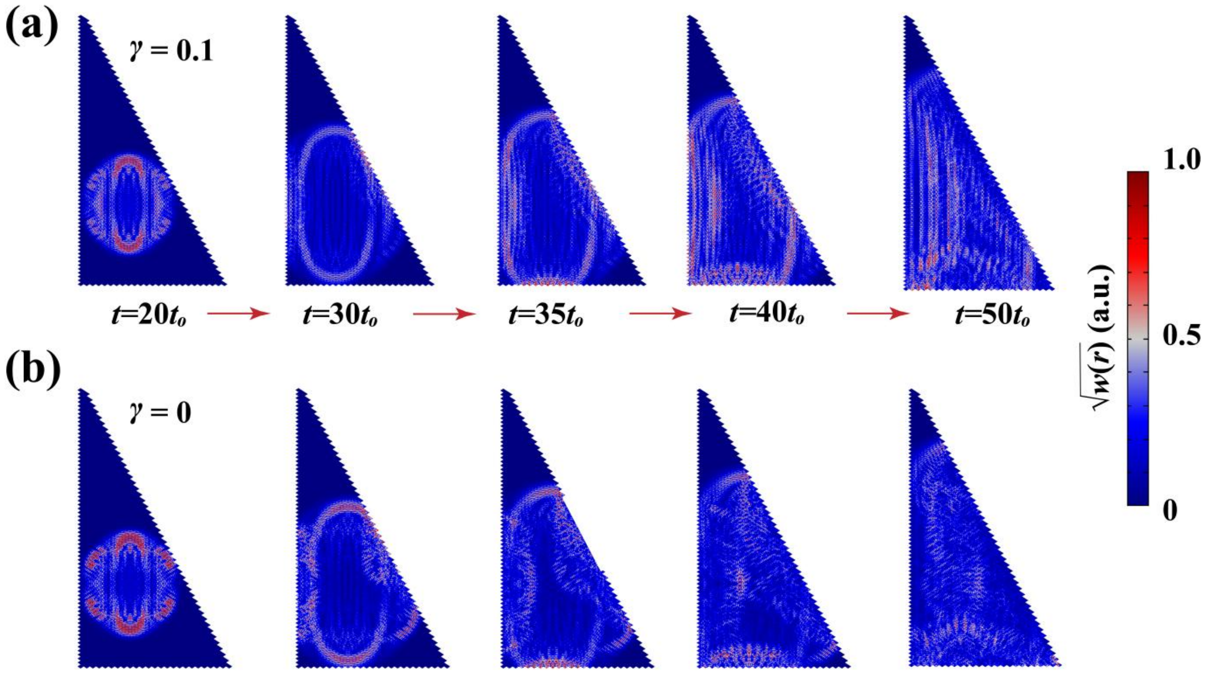

Figure S8. The dynamic evolution of a Gaussian pulse inside a 2D triangle for (a) $\gamma = 0.1$ and (b) $\gamma = 0$. Strong specular reflection is seen for the oblique edge of $\gamma = 0$, with a clear wavefront at $t = 30t_0$ and $t = 35t_0$, while strong interference with the reflection field of the vertical edge is shown at $t = 40t_0$ and $t = 50t_0$. All the parameters are the same with Figure 4 in the main text.

Section VI. Detailed analysis of the power flux in the triangular configuration

To analyze the power flux shown in Figure 4(c) of the main text more fairly, we move the source to the point marked by O in Figure S9(a) and record the waves at p, q, s, and t. To quantify the locations, we denote the unit cell in the bottom left of the triangle as (1,1), and then any unit cell can be denoted as (*m*, *n*). The first (second) letter in the parenthesis denotes the distance of the unit cell with respect to (1,1) in the horizontal (vertical) direction. With such notation, the location of the excited spot O is (5, 6), while the position of p, q, s, and t are (1, 37), (9, 37), (16.5, 10.5), and (16.5, 1.5), respectively. Since the edge of the realistic structure is formed in a zigzag way, we calculate the average power flux for the minimum period, namely four unit cells near the spots, as shown in the left and top right panel of Figure S9(a) for the spot p and q. Note that the spots p and q are symmetric about the spot O in the horizontal direction, while the spots s and t are symmetric about the spot O in the vertical direction. Therefore, the spots p and q (s and t) are treated as one pair of detecting points. We deliberately choose these points to make the comparison fair because the distances between the points in each pair and the source O are the same. Since the unit cell possesses mirror

symmetry about the x and y axes, the optical length from the source to the detecting points in each pair is also the same, and the ensuing radiation power directly from the source shall be the same.

Figures S9(b) and S9(c) respectively show the total power flux perpendicular to the interface $S_\perp$ at p/q and s/t. Besides the directly radiated waves from the source, the total waves recorded at the detecting spots also have contributions from the potential skin modes and the reflected waves from the interface. However, the features of $S_\perp$ from these two contributions are distinct. Since the skin effect occurs when the incident wave is not reflected specularly, $S_\perp$ shall be always towards the edge, but for a conventional interface with reflective waves, $S_\perp$ can be either towards or away from the edge. As a result, examining $S_\perp$ near the interface offers a simple way to identify the skin effect. The comparison between the blue and black markers in Figures S9(b) and S9(c) nicely shows the above feature and thus confirms the observation of GDSE.

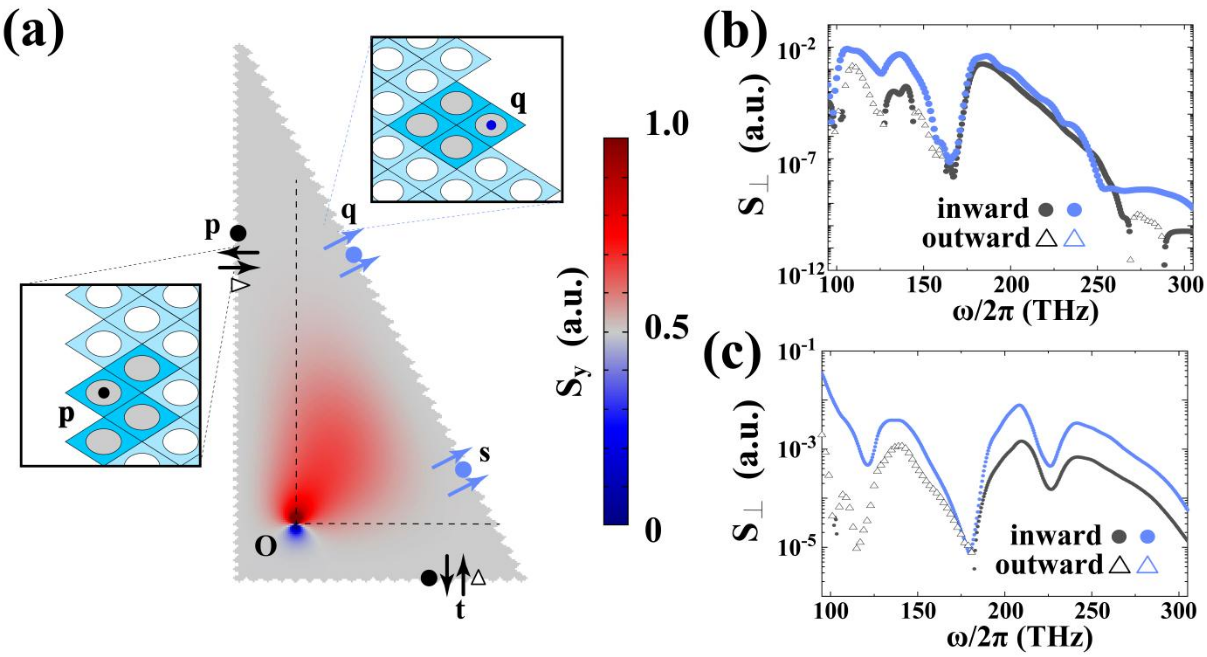

Figure S9. (a) A scheme of the calculation setup with the color plot showing the power flux along the y axis. The spot O indicates the position of the incident source, while p, q, s, and t are the detecting spots. (b) The calculated power flux perpendicular to the edges $S_\perp$ at the p (black) and q (blue). (c) The calculated power flux perpendicular to the edges $S_\perp$ at the s (blue) and t (black). The dots (triangles) indicate that the power flux is towards (away from) the edge.

References

[S1] H. Zhou, C. Peng, Y. Yoon, C. W. Hsu, K. A. Nelson, L. Fu, J. D. Joannopoulos, M. Soljacic, and B. Zhen, Observation of bulk Fermi arc and polarization half charge from paired exceptional points, Science, 359, 6379 (2018).
