# Supplementary figures and images for: Geometry-dependent skin effects in reciprocal photonic crystals
Source: Nanophotonics. 2022 Jun 20;11(15):3447–56. doi: 10.1515/nanoph-2022-0211 (PMC11501693; doi:10.1515/nanoph-2022-0211)

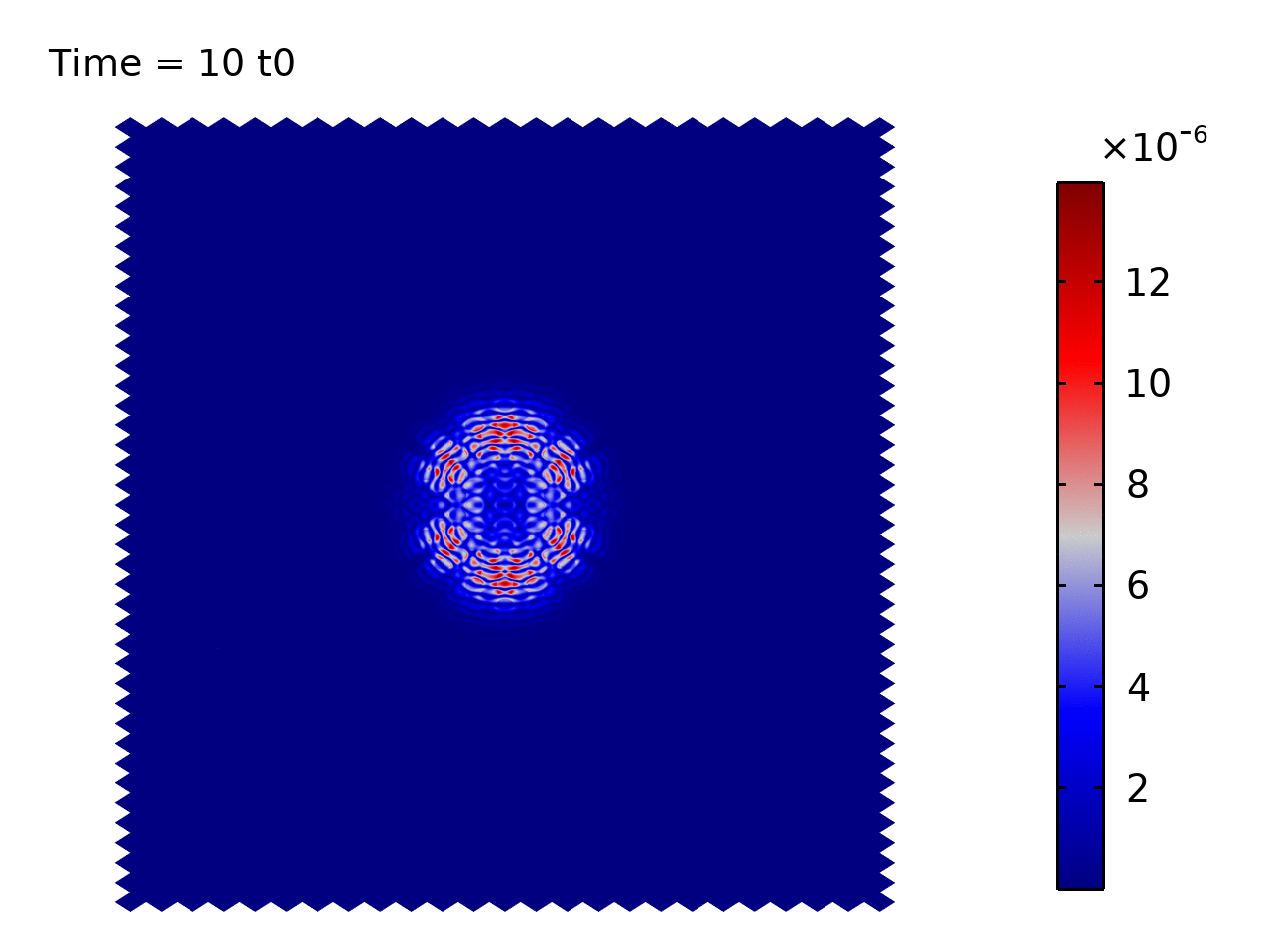

Supplement: Supplementary file 2 — Supplementary Material Details [file j_nanoph-2022-0211_suppl_002.gif]

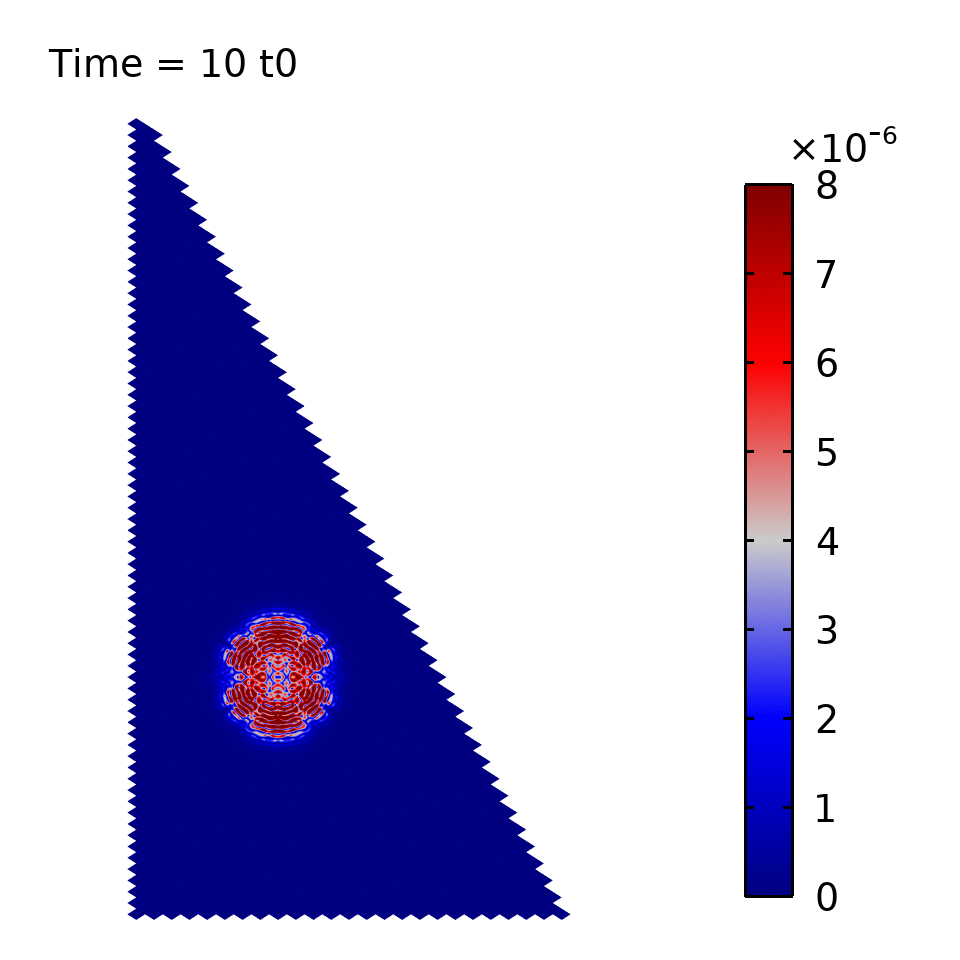

Supplement: Supplementary file 3 — Supplementary Material Details [file j_nanoph-2022-0211_suppl_003.gif]

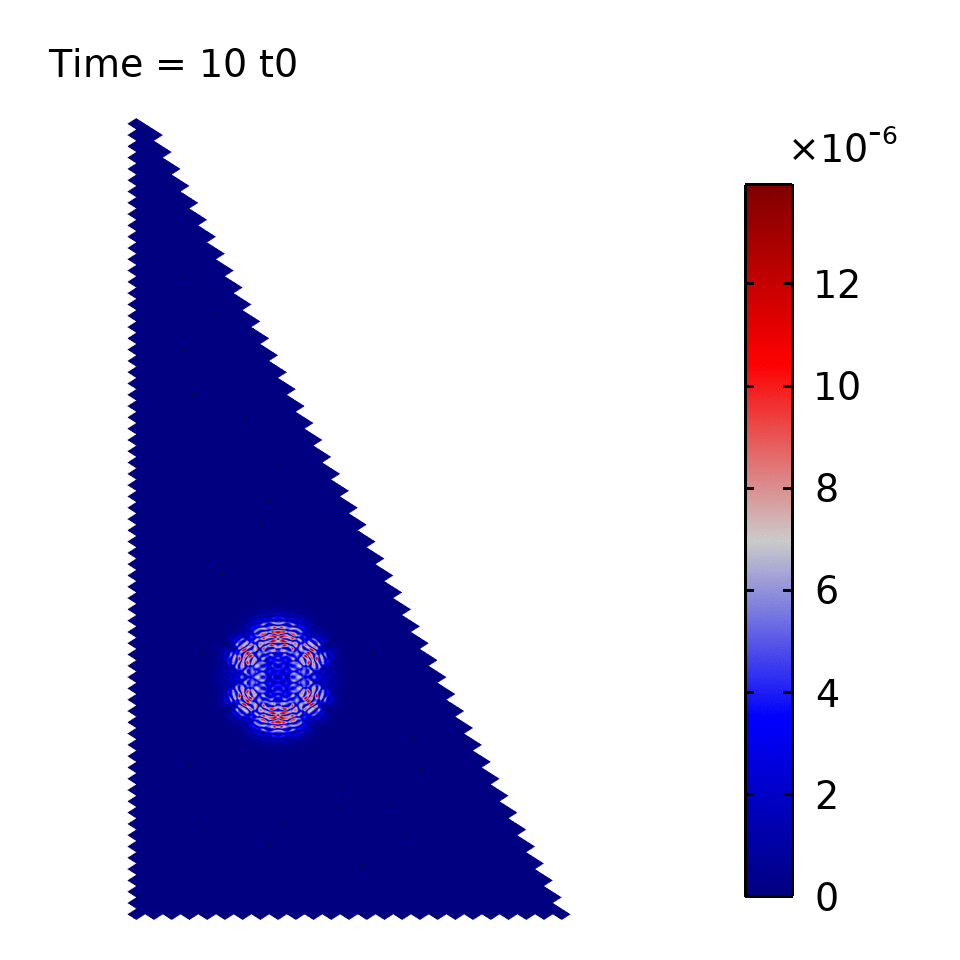

Supplement: Supplementary file 4 — Supplementary Material Details [file j_nanoph-2022-0211_suppl_004.gif]
